# Clinical and Radiographic Evaluation of Marginal Bone Level (BL) in Two Implant-Retained Mandibular Overdenture With Lingualized Occlusion (LO): A Six-Year Clinical Trial

**DOI:** 10.7759/cureus.42810

**Published:** 2023-08-01

**Authors:** Urwashi Kumari, Afreen Kouser, Asfiya Shaik, Sindhumati J, Vidhya J, Ritu Yadav

**Affiliations:** 1 Prosthodontics, Crown and Bridge, Sri Rajiv Gandhi College of Dental Sciences and Hospital, Bengaluru, IND; 2 Oral Pathology and Microbiology, Sri Rajiv Gandhi College of Dental Sciences and Hospital, Bengaluru, IND; 3 Dentistry, Manipal College of Dental Sciences, Mangalore, IND

**Keywords:** marginal bone level, lingualized occlusion, overdenture, ball attachment, dental implant

## Abstract

Background: The objective of this research was to assess the marginal bone loss and stability in a lingualized occlusal scheme with implant-supported mandibular overdentures as a viable solution for individuals experiencing difficulties with the retention of conventional mandibular dentures. The study assessed the marginal bone level (BL) using radiographic evaluation and clinically by evaluating periodontal health using probing depth index values over a period of 6 years.

Materials and Methods: Ten completely edentulous male patients with a bone height (BH) of 15-25 mm at the mandibular symphyseal region and dissatisfaction with their mandibular conventional complete denture were included in the study. Patients were willing to accept the conditions of the study and provide informed consent. Bleeding index, plaque index (PI), probing depth, and crestal BL were accessed during the follow-up period. Marginal BLs using Wical and Swoop Analysis method were evaluated at baseline (BL) (during loading), 3 years and 6 years post-loading.

Results: During the observation period, there was no incidence of implant loss, and all patients expressed contentment with the retention, stability, chewing functionality, and esthetic appeal of their dentures. Marginal BLs through clinical and radiographic evaluation, periodontal health using bleeding on probing (BOP), and probing depth were assessed during the follow-up period. Throughout the entire period of observation, no instances of implant displacement were detected.

Conclusion: The study found that the use of a lingualized occlusal scheme with two implant-retained mandibular complete overdenture effectively transmitted horizontal loads, reduced stress on individual implants leading to decreased bone loss, and increased stability.

## Introduction

Prosthodontic research targets edentulous elders, aiming to restore satisfactory esthetics and physiological functions such as speech, deglutition, and mastication. Conventional complete denture treatment has been found to improve the quality of life in such patients, but the resorbed ridge condition can cause discomfort and problems with retention and stability of the denture prosthesis, impacting facial esthetics, chewing, and biting [1]. Pre-prosthetic surgery, such as vestibuloplasty, has been traditionally used to treat such patients by extending the height of the alveolar ridge. In recent years, implant-retained overdentures have become a popular treatment option for edentulous mandible patients, with extensive research conducted on their application [2-5].

The preferred treatment option for edentulous patients, as per the McGill consensus statement, is mandibular two-implant overdentures [6]. Overdentures have been analyzed in multiple studies, and techniques, outcomes, and clinical considerations related to their use have been extensively discussed. Various treatment options are available for such patients, with the objective of enhancing their satisfaction and performance. The options consist of implant-supported mandibular overdentures that employ bar-clip attachments, ball attachments, and magnets for retaining the mandibular overdenture [6-8].

The biomechanical responses of osseointegrated implants differ from those of natural teeth due to the absence of the periodontal ligament. As a result, dental implants are more vulnerable to occlusal overload, which can lead to bone loss around the implant and failure of the prosthesis/implant. To reduce these risks, various factors such as the occlusal scheme must be taken into account. In order to achieve occlusal balance for implant-supported prostheses, it is necessary to ensure the stability of the intercuspal position and reduce lateral forces [9-10].

Different occlusal patterns have been employed in creating complete denture prostheses. Gysi first introduced the biomechanical advantages of lingualized tooth forms that included a single, linear cusp in each maxillary tooth which fit into a shallow mandibular depression. These teeth had a relatively pleasing appearance, were easy to position, and enabled vertical force transfer owing to their mortar-and-pestle structure [11]. Subsequently, Payne proposed the concept of lingualized occlusion (LO). Both in vitro and in vivo studies have shown that patients with complete dentures arranged with LO may experience superior stability. The aim of LO is to stabilize the prosthesis and slow down the rate of mandibular ridge resorption [12].

This study evaluates marginal BL clinically and radiographically and implant percussion in two-piece implant retained OD with LO in a regular interval of 0, 3, and 6 years. The present study was undertaken with the following aims and objectives to assess marginal BL in two implants retained mandibular overdenture using clinical and radiographic evaluation in a mean interval of 0, 3, and 6 years and to assess periodontal health using bleeding on probing and probing depth index values in two implants retained mandibular overdenture in a mean interval of 0, 3, and 6 years.

## Materials and methods

**Figure 1:**
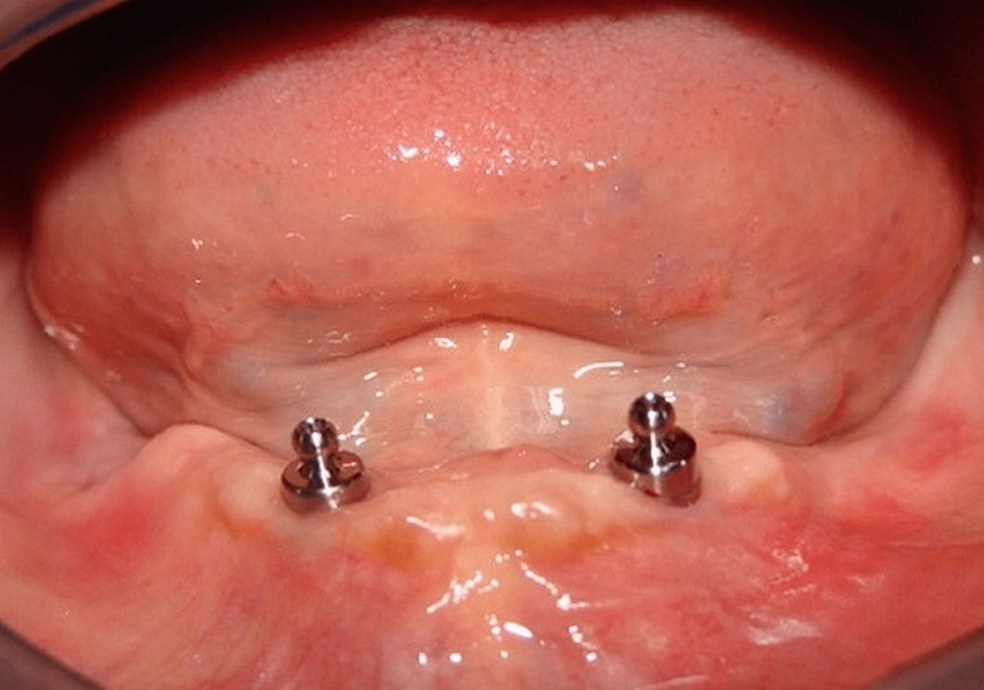
Placement of ball attachment.

**Figure 2:**
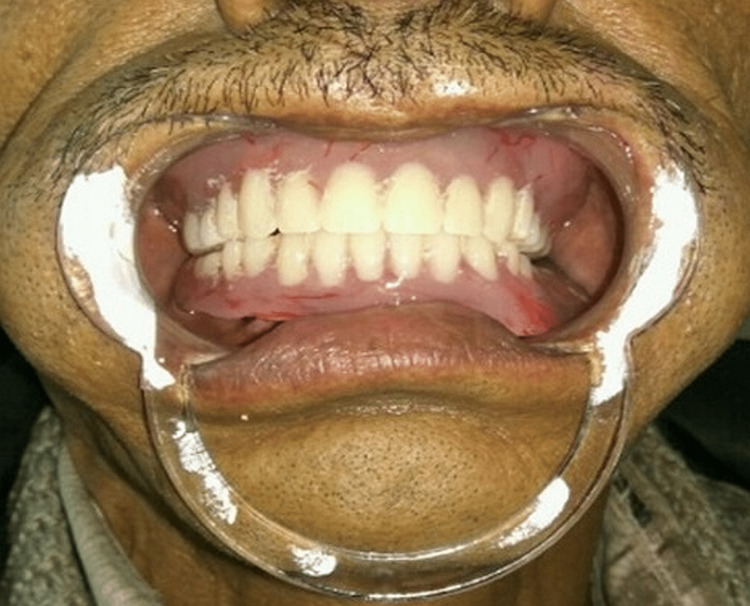
Patient with LO denture. LO, lingualized occlusion

This study involved 10 male patients who were completely edentulous and had been using conventional complete dentures for 6 months but were dissatisfied with their mandibular dentures. The study IRB number was DJD/IEC/2016/A-041. They were systemically healthy. Two-piece titanium dental implants were inserted in the mandibular inter-foraminal region of each patient and left submerged and unloaded for 5 months. After this period, the implants were exposed and healing abutments were placed for a week, simultaneously primary impression was recorded followed by border molding with heavy body elastomeric impression material and a final impression with medium body elastomeric impression material. Jaw relation was recorded and teeth arrangement following a lingualized occlusal scheme was arranged. Later, a denture trial was done and was acrylized. The mandibular denture was adjusted with an implant and a nylon cap was incorporated into it (Figures 1-2).

Semi-anatomic teeth have shallower cuspal inclines, ranging from 5 to 22 degrees, and can go up to 33 degrees for teeth designed to mimic anatomical contours. The Acry Rock semi-anatomic teeth set was used to achieve lingualized occlusion (LO), with grinding of posterior teeth to allow only the lingual cusps of upper posterior teeth to make contact with the central fossae of lower posterior teeth. The curve of Wilson was minimized or eliminated in this arrangement. The first premolars were conventionally arranged, as the first mandibular premolar has more of a cuspid's scissor function. The number of occlusal contacts is reduced in LO, with only one "centric stop" between the upper and lower posterior teeth. A wax try-in is used to verify the denture teeth arrangement for esthetics, phonetics, and jaw relations. The patient is then provided with a conventional maxillary complete denture and mandibular overdenture with ball-and-socket attachments. 

**Figure 3:**
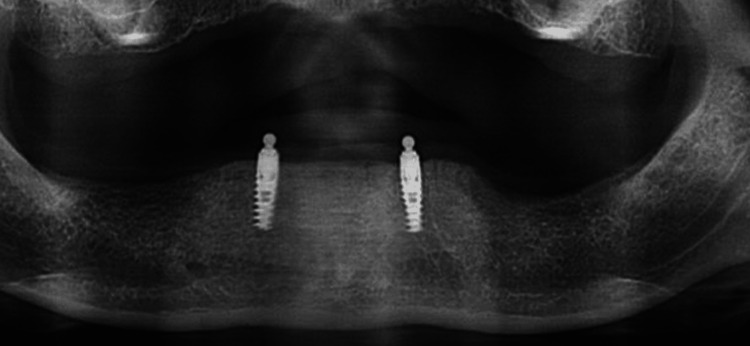
Radiograph after 3 years.

**Figure 4:**
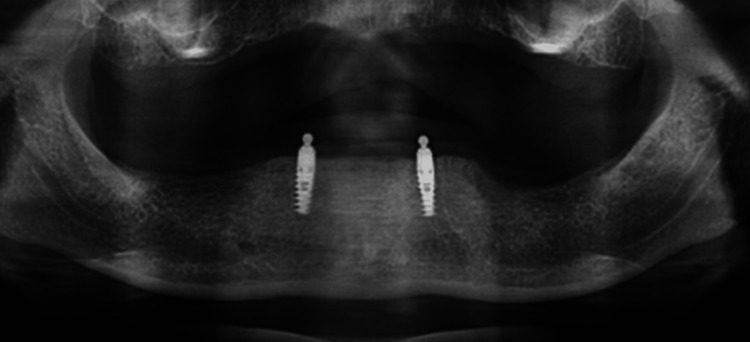
Radiograph after 6 years.

The research study investigated the clinical and radiographic evaluations of patients who were provided with overdentures. The assessments were carried out immediately after the placement of overdentures and at regular intervals of 3 and 6 years (Figures 3-4).

The clinical parameters assessed in this study included measuring probing depth using the Russell Periodontal index, evaluating the gingival bleeding index with a modified bleeding index, assessing the plaque index (PI), recording the percussion index, and documenting the mobility index. Crestal BL was assessed with an orthopantomogram (OPG) using the same machine for each patient with mental foramen as a reference point. Wical and Swoop Analysis method was used for assessing the crestal bone loss. 

**Figure 5:**
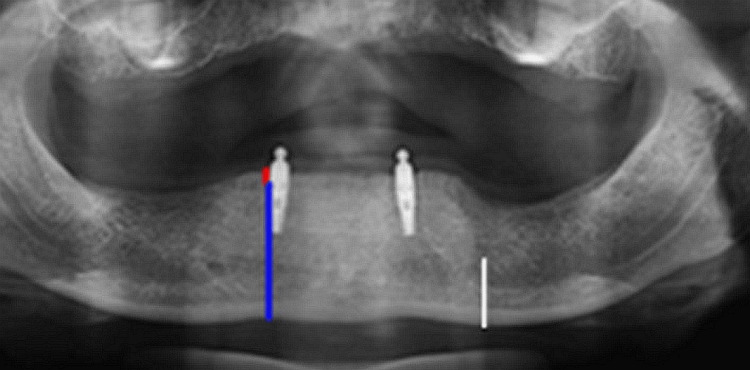
OPG showing the blue, red, and white line. Blue line represents length from crestal bone to the base of mandible (L). Red line represents the rate of resorption (R). White line represents the length from mental foramen to the base of mandible (x). OPG, orthopantomogram

The blue line on the OPG represents the length from crestal bone to the base of the mandible (L), red line represents the rate of resorption (R), and the white line represents the length from the mental foramen to the base of the mandible (x) and rate of bone resorption (R) = 3x-L (Figure 5).

**Figure 6:**
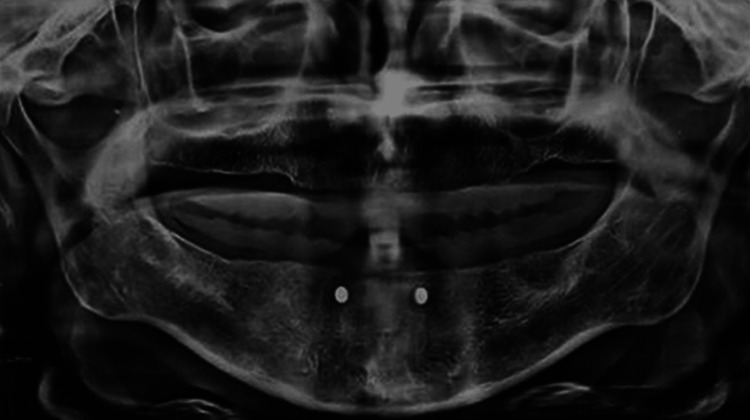
OPG with placement of steel balls. OPG, orthopantomogram

The conventional mandibular denture of the patient was temporarily converted into a radiographic template, by attaching two 4-mm diameter stainless steel balls bilaterally in the 33 and 43 regions, lingual to the teeth. Subsequently, an OPG was done with the template in place, and the actual bone height (BH) was calculated by comparing the actual and obtained dimension of the ball radiographically. This was used to check the radiograph magnification (Figure 6).

The collected data were analyzed using statistical methods including one-way (ANOVA) and t-tests, considering the follow-up period as the variable. The statistical analysis was performed using GraphPad Prism-4 software (GraphPad Software, Boston, MA) for Windows in order to ensure originality.

## Results

**Figure 7:**
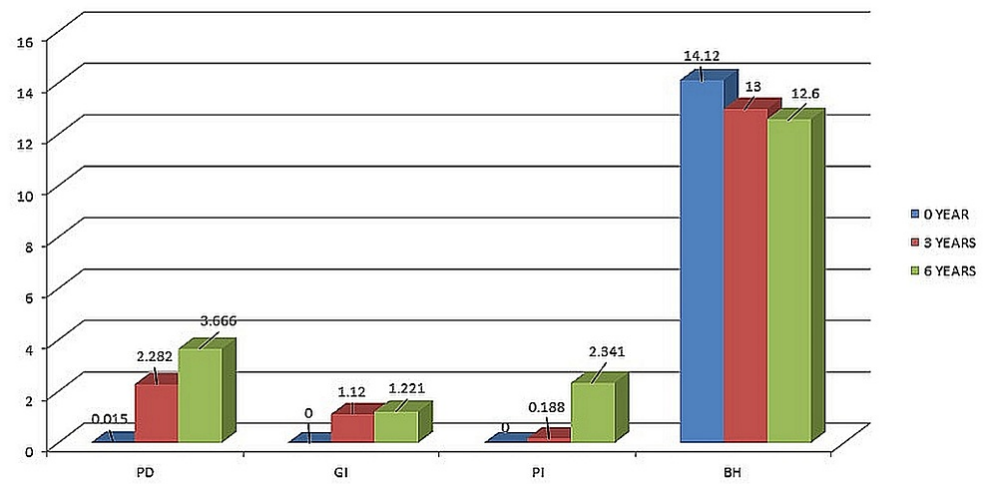
Comparison of results of PD, GI, PI, and BH during the follow-up period of 6 years. PD, pocked depth; GI, gingival index; PI, plaque index; BH, bone height

 Figure 7 shows the comparison of results of pocked depth (PD), gingival index (GI), PI, and BH during the follow-up of 6 years.

All participants reported high levels of satisfaction regarding the stability, retention, and esthetics of their dentures. Implant percussion tests consistently indicated a strong percussion sound (Score 0), with no observed movement in the implants throughout the follow-up period. During the entire follow-up period, no implant mobility was detected. The bleeding index, PI, probing depth, and crestal BL revealed significant differences fixed time points (Table 1).

**Table 1:** Mean and SD at 0 years, 3 years, and 6 years time-point. SD, standard deviation; PD, pocket depth; GI, gingival index; PI, plaque index; BH, bone height

S.NO.	Parameters	At 0 Years	At 3 Years	At 6 Years
1	Probing PD	1.042 ± 0.32	1.379 ± 0.394	1.688 ± 0.73
2	GI	0.0	0.515 ± 0.17	0.816 ± 0.32
3.	PI	0.0	0.817 ± 0.3	1.261 ± 0.37
4.	BH	13.85 ± 0.08	13.35 ± 0.14	13 ± 0.14

The mean deviation value of probing depth at the time of insertion was 0.015. After 3 and 6 years of follow-up, the mean deviation values increased to 2.282 and 3.666, respectively. Statistical analysis using paired sample t-tests revealed significant differences in probing depth mean values between the 3-year and BL measurements, the 6-year and BL measurements, as well as between the 6-year and 3-year measurements (Table 2).

**Table 2:** Comparison between successive time-intervals (by paired “t” test), % improvement between time-points for probing PD. PD, pocket depth

S.no.	Difference between time-intervals	Mean ± SD (differences)	%Improvement between time points	Probability of paired “t” test	p-Value
1	0 years-3 years	-0.93 ± 0.15	80.86	0.001	p<0.05
2	3 years-6 years	-0.25 ± 0.05	11.96	0.001	p<0.05

The result of the paired sample t-test of the probing depth mean values revealed significant differences from 3 years to BL, 6 years to BL, and from 6 to 3 years.

For the GI, the mean and SD values at BL, 3 years, and 6 years were 0.25, 1.12, and 0.13, respectively. Paired sample t-tests (the study was designed to compare two groups mainly that is 0 years-3 years and 3 years-6 years) showed significant differences in GI mean values between 3-year and BL measurements, as well as between the 6-year and BL measurements. However, there was no significant difference in GI mean values between the 3-year and 6-year measurements (Table 3).

**Table 3:** Comparison between successive time-intervals (by paired “t” test), % improvement between time-points for GI. GI, gingival index

S. NO.	Difference between time-intervals	Mean ± SD (differences)	%Improvement between time-points	Probability of paired “t” test	p-Value
1	0 year-3 years	0.25 ± 0.12	20.49	0.001	p<0.05
2	3 years-6 years	0.13 ± 0.07	13.4	0.001	p<0.05

The result of the paired sample t-test of the GI mean values revealed significant differences from 3 years to BL and 6 years to BL while there was no significant difference from 3 years to 6 years.

Regarding the PI, the mean deviation values at BL, 3 years, and 6 years were 0.0, 0.188, and 2.341, respectively. Paired sample t-tests indicated significant differences in PI mean values between the 6-year and BL measurements, as well as between the 6-year and 3-year measurements. No significant difference was observed between the 3-year and BL measurements (Table 4).

**Table 4:** Comparison between successive time-intervals (by paired “t” test), % improvement between time-points for PI. PI, plaque index

S.no.	Difference between time intervals	Mean ± SD (differences)	% improvement between time points	Probability of paired “t” test	p-Value
1	0 year-3 years	-0.61 ± 0.05	49.14	0.001	p<.05
2	3 years-6 years	-0.26 ± 0.08	13.97	0.001	p<.05

The result of the paired sample t-test of the PI mean values revealed significant differences from 6 years to BL and 6 to 3 years while there was no significant difference from 3 years to BL.

In terms of BH, the mean deviation values at insertion, 3 years, and 6 years were 14.12, 13.0, and 12.6, respectively. Paired sample t-tests for the mesial lateral (ML) BH demonstrated significant differences between the BL and 3-year measurements, the 3-year and 6-year measurements, as well as between the BL and 6-year measurements (Table 5).

**Table 5:** Comparison between successive time intervals (by paired “t” test), % improvement between time-points for bone loss.

S.no.	Difference between time intervals	Mean ± SD (differences)	% improvement between time points	Probability of paired “t” test	p-Value
1	0 year	0.45 ± 0.05	45	0.001	p<.05
2	3 years	0.19 ± 0.02	42.22	0.001	p<.05
3	6 years	0.09 ± 0.02	14.06	0.001	p<.05

The result of the paired sample t-test of the BH mean values for ML revealed significant differences from BL to 3 years, from 3 years to 6 years, and from BL to 6 years.

## Discussion

Implant-supported overdentures present numerous practical advantages compared to traditional complete dentures and removable partial dentures. These advantages encompass decreased bone resorption, enhanced esthetics, improved tooth positioning, optimized occlusion, better preservation of the occlusal vertical dimension, and minimized prosthesis movement [13]. Furthermore, implant-supported overdentures contribute to improved phonetics, enhanced psychological well-being, and an overall better quality of life for patients. Unlike traditional dentures, which rely on the residual alveolar ridge and mucosa for support and retention, implant-supported overdentures demonstrate superior retention. Patients consistently express greater satisfaction with implant-supported overdentures, irrespective of the type of attachment system employed (e.g., bar, ball, or magnet). These overdentures offer increased stability and comfort, allowing individuals to consume a broader range of foods and enhance their overall nutritional well-being. Patients also report easier speech with implant-supported overdentures [14]. Mandibular overdentures using two implants can be a cost-effective option when compared to multiple implant-supported overdentures. In order to choose the appropriate implant site for these overdentures, factors such as bone volume, quality, and amount, as well as the anterior arch's size and curvature, should be taken into consideration.

Implant-supported overdentures are more stable than traditional dentures due to the mechanical attachment of the implant support system. Traditional mandibular dentures have the tendency to move up to 10 mm during function, which can make it challenging to control occlusion and masticatory forces. In the field of dentistry, researchers have consistently sought an ideal artificial tooth arrangement that offers denture stability, comfort, esthetics, and functionality. Among the various approaches, one widely accepted scheme is known as LO, which combines anatomical and mechanical principles. This concept leverages the benefits of both anatomical and non-anatomical occlusion to achieve optimal outcomes [15-16].

The origins of LO can be traced back to Alfred Gysi, who first introduced the concept in 1927 with his work on "Cross-Bite Posterior Teeth." Subsequently, S.H. Payne refined the concept in 1941, attributing its development to Farmer. The term "lingualized occlusion" was later coined by Pound (references). Over the years, substantial modifications have been made to the idea of LO, leading to enhanced stability of dentures and greater comfort for patients. This type of occlusion provides a balanced arch, where the maxillary teeth's lingual cusps are the only points of contact with the mandibular posterior teeth, minimizing horizontal pressure and redirecting vertical forces onto the mandibular residual ridges [17-18]. An LO is a viable option for denture combinations, especially in cases where anatomic occlusal schemes are not possible due to severe alveolar resorption, a Class II jaw relationship, or displaceable supporting tissue. The utilization of this occlusal scheme provides improved esthetic results while preserving the advantages of a non-anatomic system. It proves beneficial in scenarios where a complete denture opposes a removable partial denture, particularly in cases like combination syndrome where bilateral balanced occlusion is desired. Additionally, LO is recommended for patients with implant-supported overdentures who have parafunctional habits, as it reduces the number of horizontal forces exerted on supporting tissues.

The achievement of dental implants is significantly influenced by the occlusal relationship of the implant-supported overdenture. In this investigation, two implants were placed in the anterior mandible, specifically in the region between the mental foramina, which is known for its favorable bone quality and quantity to support implants. This anatomical area is characterized by dense cortical plates and trabecular bone. The overdentures were attached to the implants using a ball-and-socket mechanism that allows for multidirectional movement [19]. Based on the available literature, it has been observed that occlusal contacts on buccal cusps can generate offset and angled loads on the ridge, leading to potential complications. To mitigate this issue, a median lingualized occlusal scheme was employed, where primary occlusal contacts were restricted within the implant diameter and central fossa, while secondary contacts were limited to within 1 mm of the implant periphery to minimize moment loads.

The objective of this study was to assess probing PD, GI, PI, and bone loss. The findings revealed statistically significant variations in mean probing depth throughout the study duration, consistently measuring less than 1 mm. These outcomes align with previous research indicating that probe penetration of approximately 3 mm indicates successful implants, while deeper pockets may suggest inflammation at the base of the defect [20].

During the span of a year, noticeable disparities in gingival and index scores were detected, indicating the presence of mild gingival inflammation. These outcomes align with the research conducted by Behneke et al. [21], which suggests that individuals may encounter difficulties in upholding optimal oral hygiene practices, resulting in a rise in visible plaque accumulation and gingival inflammation. There was no indication of any movement in any of the examined implants throughout the follow-up period of the study. The mobility index score remained at zero, which suggests that the implants had excellent initial stability that resulted in effective osseointegration and enhanced anchorage to the neighboring bone. The presence of a solid ringing sound during percussion also served as evidence of successful osseointegration [22].

Determining the exact threshold for bone loss that signifies the success or failure of dental implants can be a complex task. However, it is widely accepted that a loss of more than 50% of the implant's crestal bony contact height poses a significant risk and indicates implant failure, regardless of the initial implant-bone contact. The evaluation of crestal bone plays a crucial role in assessing the overall health of dental implants. In this particular study, we observed a minor degree of crestal bone loss after 3 years in all of the implants. After a six-year period, the average loss of peri-implant marginal bone was found to be statistically significant, measuring 0.5 ± 0.13 mm for all implants. These findings align with the results reported by Ismail et al., who also documented comparable levels of marginal bone loss [17].

During the observation phase, a higher amount of bone loss was detected in the initial 6-month period than in the following 6 years (Wical and Swoop Analysis method). This pattern is consistent with Misch's research, which indicates that greater crestal bone loss occurs during the early phase of healing. However, it should be noted that the loss of bone in this context can be attributed to the natural bone modeling and remodeling that occurs after surgery. This process is influenced by the mechanical strain exerted on the implant, which allows the implant to adapt and integrate with the surrounding biomechanical environment. The absence of mobility change could be attributed to appropriate prosthesis design. Previous research has demonstrated that appropriate design enables effective transmission of horizontal loads, reducing the stress on individual implants, enhancing stability, and improving chewing ability. Consequently, based on the outcomes of this study, the selected occlusal scheme was regarded as successful [23].

The study has some limitations, like as the questionnaire being dependent on participant preference, the limited number of participants may restrict the generalizability of the findings, and limit the statistical power of the analysis. The study evaluated the outcomes over a period of 6 years and determined that the chosen occlusal scheme was effective. While this duration allows for an assessment of medium-term results, long-term data would provide more comprehensive insights into the stability, bone maintenance, and patient satisfaction with the overdentures. The study included only male patients, which may limit the generalizability of the findings to female patients or diverse populations. The inclusion of both genders would have enhanced the external validity of the study. Multi-center studies involving different populations and settings would strengthen the validity and generalizability of the findings.

## Conclusions

As per the results of the mean marginal bone loss and the other peri-implant parameters after 6 years of loading, the implant retained overdenture with median lingualized occlusal schemes may be recognized as being acceptable according to the general implant success rates and criteria. The use of LO in clinical dentistry is not a passing fad, but rather a crucial element of effective practice. This approach serves to decrease the pressure on denture-bearing areas, improve stability, and provide a solution for the use of proper prosthesis design could account for the lack of mobility change as it has been shown that proper design is effective in transmitting the horizontal loads, by their reducing the stress placed on individual implants for clinical challenges. In general, there are no opposing factors for utilizing LO. A recent study examined a two-implant supported mandibular complete overdenture with a lingualized occlusal scheme for treating a resorbed mandibular ridge. The results showed that the treatment had a low rate of bone resorption and successfully enhanced retention due to the implants and increased stability from the LO. Appropriate prosthesis design can help minimize stress on individual implants and improve masticatory function.

Therefore, when creating complete denture occlusion, it is crucial to consider all factors that promote denture base stability, including biological, physiological, and mechanical factors, and to prevent excessive force on the underlying structures. In the treatment of denture occlusion, the preservation of supporting structures should be given top priority.
